# Epigenome-wide DNA methylation profiling of periprostatic adipose tissue in prostate cancer patients with excess adiposity—a pilot study

**DOI:** 10.1186/s13148-018-0490-3

**Published:** 2018-04-17

**Authors:** Yan Cheng, Cátia Monteiro, Andreia Matos, Jiaying You, Avelino Fraga, Carina Pereira, Victoria Catalán, Amaia Rodríguez, Javier Gómez-Ambrosi, Gema Frühbeck, Ricardo Ribeiro, Pingzhao Hu

**Affiliations:** 1 Department of Biochemistry and Medical Genetics & Department of Electrical and Computer Engineering, University of Manitoba, Winnipeg, Canada; 2 Experimental Center, Northwest University for Nationalities, Lanzhou, People’s Republic of China; 3 Molecular Oncology Group, Portuguese Institute of Oncology, Porto, Portugal; 4 Research Department, Portuguese League Against Cancer–North, Porto, Portugal; 5 Laboratory of Genetics and Environmental Health Institute, Faculty of Medicine, University of Lisboa, Lisbon, Portugal; 6 Tumor & Microenvironment Interactions, i3S/INEB, Institute for Research and Innovation in Health, and Institute of Biomedical Engineering, University of Porto, Porto, Portugal; 7 Department of Urology, Centro Hospitalar Universitário do Porto, Porto, Portugal; 8 CINTESIS, Center for Health Technology and Services Research, Faculty of Medicine, e, University of Porto, Porto, Portugal; 9 Metabolic Research Laboratory, Universidad de Navarra, Pamplona, Spain; 10 CIBER Fisiopatología de la Obesidad y Nutricion, Instituto de Salud Carlos III, Madrid, Spain; 11 Department of Endocrinology, Clínica Universidad de Navarra, Pamplona, Spain; 12 Department of Clinical Pathology, Centro Hospitalar e Universitário de Coimbra, Coimbra, Portugal; 13 i3S/INEB, Instituto de Investigação e Inovação em Saúde/Instituto Nacional de Engenharia Biomédica, Universidade do Porto, Tumor & Microenvironment Interactions, Rua Alfredo Allen, 208 4200-135 Porto, Portugal

**Keywords:** DNA methylation, Periprostatic adipose tissue, Obesity, Prostate cancer, Microenvironment

## Abstract

**Background:**

Periprostatic adipose tissue (PPAT) has been recognized to associate with prostate cancer (PCa) aggressiveness and progression. Here, we sought to investigate whether excess adiposity modulates the methylome of PPAT in PCa patients. DNA methylation profiling was performed in PPAT from obese/overweight (OB/OW, BMI > 25 kg m^−2^) and normal weight (NW, BMI < 25 kg m^−2^) PCa patients. Significant differences in methylated CpGs between OB/OW and NW groups were inferred by statistical modeling.

**Results:**

Five thousand five hundred twenty-six differentially methylated CpGs were identified between OB/OW and NW PCa patients with 90.2% hypermethylated. Four hundred eighty-three of these CpGs were found to be located at both promoters and CpG islands, whereas the representing 412 genes were found to be involved in pluripotency of stem cells, fatty acid metabolism, and many other biological processes; 14 of these genes, particularly *FADS1*, *MOGAT1*, and *PCYT2*, with promoter hypermethylation presented with significantly decreased gene expression in matched samples. Additionally, 38 genes were correlated with antigen processing and presentation of endogenous antigen via MHC class I, which might result in fatty acid accumulation in PPAT and tumor immune evasion.

**Conclusions:**

Results showed that the whole epigenome methylation profiles of PPAT were significantly different in OB/OW compared to normal weight PCa patients. The epigenetic variation associated with excess adiposity likely resulted in altered lipid metabolism and immune dysregulation, contributing towards unfavorable PCa microenvironment, thus warranting further validation studies in larger samples.

**Electronic supplementary material:**

The online version of this article (10.1186/s13148-018-0490-3) contains supplementary material, which is available to authorized users.

## Background

Prostate cancer (PCa) is one of the most frequent malignancies in men and the second leading cause of cancer-related death in the North America and most western European countries [1, 2]. Epidemiological studies support obesity or excess adiposity as an important environmental risk factor for PCa, being primarily associated with advanced disease and death [3]. Periprostatic adipose tissue (PPAT), a white fat depot surrounding the prostate capsular-like structure, has been recognized to have the potential to exert pro-tumoral endocrine and paracrine influences on prostate cancer cell’s biological phenotypes [4]. There is now evidence that obesity and overweight result in excess fat deposit at PPAT [5], altered fatty acid profile [6], migration of tumor cells [7], secretion of a variety of adipokines, such as interleukin-1 beta (IL-1b), osteopontin, leptin, tumor necrosis factor alpha (TNF-a), and decreased adiponectin, thus contributing to a tumor microenvironment that ultimately facilitates PCa aggressiveness [7, 8].

DNA methylation is a well-known epigenetic mechanism resulting from the interaction between environmental factors and the genome [9]. DNA methylation with variation of CpG sites is associated with tissue-specific gene modulation and involved in phenotype transmission and in the development of diseases [10]. Excess adiposity, as a consequence of environmental factors such as excessive food consumption or inactive lifestyle, has been identified as a regulator of epigenetic modification in adipose tissue. Recent findings from experimental studies suggested that modification of DNA methylation pattern in adipose tissue and adipocytes was related with development of cancer, type 2 diabetes, and cardiovascular diseases through influencing metabolism and inflammation [11–13]. Additionally, several studies reported altered DNA methylation in PCa cells as compared with adjacent benign tissue, and some significantly methylated CpG sites and genes were found to be responsible for the occurrence and progression of PCa [14–16]. Nevertheless, the epigenome-wide DNA methylation profile of PPAT from excess adiposity PCa patients is currently unknown despite its potential mechanistic involvement in obesity association with PCa.

The aim of this study was to perform a epigenetic-wide association study (EWAS) in order to evaluate DNA methylation profile of PPAT obtained from obese/overweight (OB/OW) in comparison with normal weight (NW) PCa patients and identify differentially methylated sites. We also explored the consequential potential biological functions that account for the effect of PPAT from OB/OW subjects in PCa molecular mechanisms.

## Methods

### Study samples

This study included ten prostate cancer patients from the Portuguese Institute of Oncology, Porto Centre. Inclusion criteria and conditions of this study have been previously reported, including the procedures for PPAT collection, handling, and storage [4]. Briefly, PPAT was collected and immediately processed in the operating room and transported to the laboratory within 2 h in appropriate culture media and temperature conditions, in order to minimize pre-analytical errors. Patients’ signed informed consent and research procedures were approved by the institute’s ethics committee.

The clinical and pathological characteristics of participants are presented in Table 1. The ten subjects were selected from a larger group of patients undergoing prostate surgery (*n* = 51) [4, 17] that fitted the strict inclusion and exclusion criteria, in order to control for variables that might influence adipose tissue gene expression or methylation (e.g., anti-diabetic or anti-dyslipidemia drugs, stage of disease and PSA, concomitant diseases such as diabetes, other neoplasia or metabolic syndrome). Subjects were matched for age at diagnosis, PSA value, Gleason grade, and stage of disease, which differed in body mass index (BMI). BMI was calculated by dividing weight in kilograms by the squared height in meters and categorized using the WHO (World Health Organization) criteria: normal weight, BMI < 25 kg m^−2^, overweight, 25 ≤ BMI < 30 kg m^−2^, and obese, BMI ≥ 30 kg m^−2^. Obese and overweight were combined into one excess adiposity group (*n* = 5, BMI≥25 kg m^−2^) versus normal weight group (*n* = 5, BMI < 25 kg m^−2^). Therefore, the two groups were selected to differ only by BMI, in order to reflect our objective of assessing whether excess adiposity (BMI) influences PPAT methylation profile.

**Table 1:** Clinicopathological characteristics of PCa patients by BMI category

Character	NW (*n* = 5)	OB/OW (*n* = 5)	*P* value
Age (years)	65.2 ± 3.8	63.2 ± 2.5	0.67^a^
BMI (kg/m^2^)	23.0 ± 0.3	29.0 ± 0.9	0.0003^a^
Gleason score			
< 7	2 (40%)	1 (20%)	
≥ 7	3 (60%)	4 (80%)	1.00^b^
Stage			
OCPCa	2 (40%)	2 (40%)	
EPCa	3 (60%)	3 (60%)	1.00^b^
Smoking status			
Yes	1	5	
No	4	0	0.05^b^
PSA (ng/ml)	10.7 ± 2.7	12.1 ± 3.23	0.74^a^

Data are presented as mean ± SD or number (%). Significant difference between OB/OW and NW was evaluated using ^a^*t* test and ^b^Fisher’s exact test

*OB/OW* obese/overweight, *NW* normal weight, *BMI* body mass index; PSA, prostate specific antigen; PCa, prostate cancer; OCPCa, organ-confined prostate cancer; EPCa, extra-prostatic PCa

### Epigenome-wide DNA methylation analysis

DNA was isolated from PPAT using Puregene hisalt extraction method (Qiagen/Gentra). Briefly, the tissue was minced with scalpels in a sterile petri dish on ice and then transferred to Puregene Cell Kit for overnight Proteinase K digest at 55 °C. A second Proteinase K digest was done the next morning for 5 h. DNA from the digested tissue was purified using Puregene extraction protocol (Qiagen/Gentra). Purified DNA was washed 2× with 70% ethanol and DNA pellet air dried and rehydrated in TE (10 mM Tris-Cl, 1 mM EDTA pH 7.5). Epigenome-wide DNA methylation was analyzed using the Infinium Human Methylation450 (HM450) BeadChip (Illumina, San Diego, CA, USA) in the Center for Applied Genomics (Toronto). This array contains 485,577 probes, which cover 21,231 (99%) RefSeq genes. Briefly, DNA was bisulfite-converted using the EZ DNA methylation kit (Zymo Research, Orange, CA, USA) and then used on the Infinium Assay® followed by the Infinium HD Assay Methylation Protocol (Illumina). The imaging data on the BeadChips was captured by Illumina iScan system.

### Data filtering and normalization

Raw methylation level for each probe was represented by methylation *β* value, which was calculated based on *β* = intensity of the methylated allele/(intensity of the unmethylated allele + intensity of the methylated allele + 100). *M* values were the logit transformation of *β* values based on *M* = log_2_ (*β*/(1 − *β*)), which makes the data more homoscedastic and appropriate for further bioinformatic and statistical analysis.

Methylation values were normalized using the functional normalization algorithm implemented in Minfi *R* package [18]. Quality control was performed by excluding CpG probes, which are found by Chen et al. to be cross-reactive with areas of the genome not at the site of interest [19], as well as control probes and probes on sex chromosomes. We analyzed a total of 438,458 CpG sites from the PPAT of 5 OB/OW PCa patients and 5 NW PCa patients.

### Differential methylation analysis

A statistical linear modeling approach was applied to the detected differentially methylated CpG sites (DMCs) associated with obesity in PPAT using the Bioconductor “limma” package [20]. Hyper- or hypomethylation was determined when methylation levels of CpGs increased or decreased between the OB/OW PCa group and the NW PCa group based on mean different *β* > 0 or < 0. False discovery rate (FDR)-corrected *P* values were determined according to the method of Benjamin and Hochberg’s (BH method) multiple testing procedure [21].

Differentially methylated regions (DMRs) were identified using the “Bumphunter” method implemented in the “chAMP” *R* package with the parameters (*B* = 1000, useWeights = TRUE, minProbes = 10, pickCutoff = TRUE, and other settings with default values) [22].

The proportions of significant hyper- or hypomethylated CpGs were calculated and visualized according to their relation to the nearest genes or to the CpG islands, separately. Gene promoter region was defined as 1500 base pairs (bp) and 200 bp upstream of the transcription start site (TSS) (TSS1500 and TSS200) [23]. Identified genes were selected when more than two significantly hypermethylated CpGs were simultaneously located in the promoter region.

### Functions, pathway, and network enrichment analysis

Gene ontology (GO) and KEGG pathway enrichment analyses were performed to explore the biological functions of significantly methylated genes using the online bioinformatic tool Enrichr [24]. Protein-protein interaction (PPI) analysis of all DMC-related genes was performed using NetworkAnalyst according to STRING database [25].

### Association analysis between DNA methylation and gene expression

We have previously performed gene expression experiment of the PPAT of the 5 OB/OW PCa patients and the 5 NW PCa patients using the HG-U133 Plus 2.0 Affymetrix GeneChip Array (Affymetrix, Santa Clara, CA, USA) [4]. Differential gene expression (DGE) analysis between the OB/OW PCa patients and the NW PCa patients was re-performed using the Bioconductor “limma” package as previously described [4]. Spearman’s rank correlation analysis was performed between the methylation profiles of the hypermethylated CpGs and the gene expression profiles of the genes in PPAT.

## Results

### Clinical characteristics

Clinical characteristics of PCa patients in this study were stratified according to obesity classification groups and are presented in Table 1. Mean age, PSA level, Gleason sum score, and cancer stage in subjects with PCa were similar (*P* value > 0.05) between OB/OW and NW groups. As expected, the mean BMI of the OB/OW group was significantly higher than that of the NW subjects (*P* value < 0.01). All the patients in the OB/OW group are ex-smokers or active smokers, while only one patient in the NW group is a smoker (*P* value = 0.05).

### Epigenome-wide DNA methylation profiling of PPAT

**Fig. 1:**
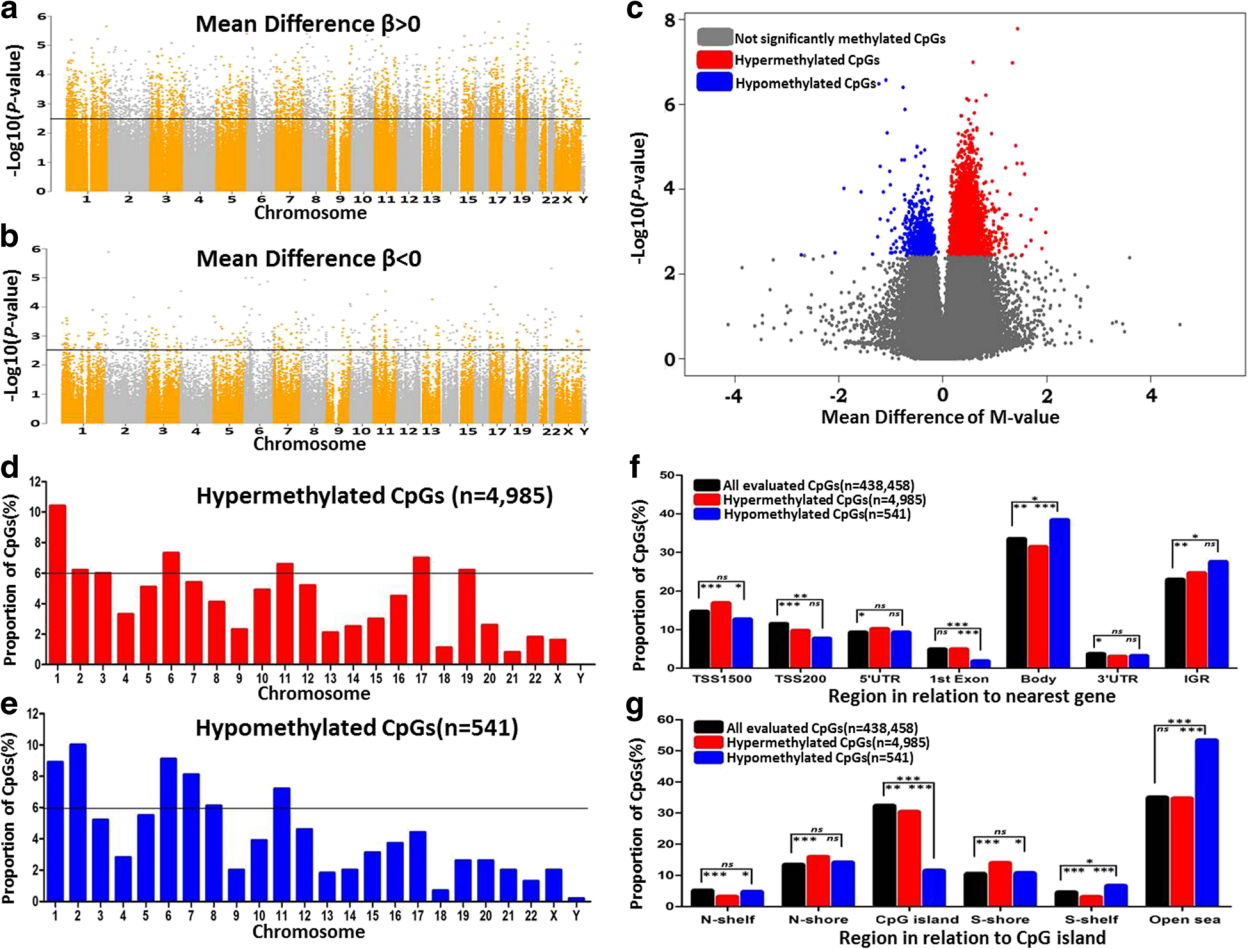
Epigenetic profiles of differentially methylated CpGs of PPAT between OB/OW and NW groups. Manhattan plots show epigenetic profiles of all increased methylated CpGs (**a**) and all decreased methylated CpGs (**b**). The *X*-axis shows chromosomes, and the *Y*-axis is a −log10 (*P* value). The black line represents the threshold of adjusted *P* value = 0.25. CpGs above the black line are significantly hyper- or hypomethylated. The volcano plot of DNA methylation (**c**) shows a significant difference in PPAT between the OB/OW and NW groups. Four thousand nine hundred eighty-five hypermethylated CpGs are labeled in red, and 541 hypomethylated CpGs are labeled in green (adjusted *P* value > 0.25). The proportions of hyper- and hypomethylated CpGs on each chromosome are shown in (**d**) and (**e**). The black line indicates if the proportions of hyper- and hypomethylated CpGs on a chromosome are higher than 6%. The distribution of significant DMCs (hyper- or hypomethylated CpGs) and globe DNA methylation CpGs in locations related to the nearest gene regions and CpG islands are shown in **f** and **g**. Hypermethylated CpGs are mainly located at TSS1500 (transcription start sites 1500), IGR (intergenic region), N-shore (the 2 kb regions upstream of the CpG island boundaries), and S-shore (the 2 kb regions downstream of the CpG island boundaries), and hypomethylated CpGs are mostly located at the gene body and open sea. The difference of the proportion of CpGs among the three CpG groups was calculated based on the *χ*^2^ test (*P < 0.05, **P < 0.01, ***P < 0.001, ns not significant). CpG islands were defined as DNA sequences (500 base windows; excluding most repetitive Alu-elements) with a GC base composition greater than 50% and a CpG observed/expected ratio of more than 0.6. The 2 kb regions immediately upstream (N_Shore) and downstream (S_Shore) of the CpG island boundaries were defined as “CpG island shores,” and the 2 kb regions upstream (N_Shelf) and downstream (S_Shelf) of the CpG island shores were referred as “CpG island shelves.” Open seas were the regions more than 4 kb from CpG islands

To study the impact of obesity status on DNA methylation profiles and to identify differentially methylated CpG sites in PPAT from OB/OW and NW prostate cancer patients, we conducted epigenome-wide DNA methylation analyses. A flowchart of the data analysis is depicted in Additional file 1: Figure S1. After quality control and filtering, the Infinium array generated methylation data for 438,458 CpG sites, from which 5526 were differentially methylated after FDR control in the PPAT of OB/OW PCa patients compared to NW (adjusted *P* value < 0.25; Additional file 2: Table S1 and Table 2). The unsupervised hierarchical clustering of DMCs showed differential DNA methylation patterns in PPAT between OB/OW and NW samples (Additional file 3: Figure S2). The majority of DMCs were hypermethylated (*n* = 4985, 90.2%), with 9.8% hypomethylated CpG sites (*n* = 541) in OB/OW versus NW prostate cancer patients (Fig. 1a, b, c).

**Table 2:** Differentially methylated CpG sites in PPAT between obese/overweight PCa patients and normal weight controls

Probe ID	Chromosome and coordinate (GRCh37)	Nearest gene	Relation to gene region	Relation to CpG island	DNAm *β* difference (%)	*P* value	Adjusted *P* value (< 0.25)
Hypermethylated CpG sites							
cg09476130	chr1:159870086	CCDC19	TSS200	Island	12.1	1.87E−03	0.213
cg21293934	chr18:14748230	ANKRD30B	TSS200	Island	11.2	1.83E−03	0.212
cg16925210	chr2:216946718	PECR	TSS200	Island	11.2	2.44E−03	0.226
cg11625005	chr5:1295737	TERT	TSS1500	Island	11.1	1.38E−03	0.196
cg07039560	chr5:140683681	SLC25A2	TSS200	Island	10.5	2.24E−03	0.222
cg00329447	chr8:145028170	PLEC1	TSS200	Island	10.1	3.41E−03	0.244
cg24463471	chr1:25257978	RUNX3	TSS1500	Island	9.9	3.58E−04	0.155
cg26149485	chr19:2428350	TIMM13	TSS1500	Island	9.7	3.36E−04	0.154
cg05156901	chr22:51016646	CPT1B	TSS200	Island	9.3	3.05E−03	0.238
cg18689454	chr21:45705694	AIRE	TSS200	Island	9.3	7.33E−04	0.174
cg01454592	chr3:49236800	CCDC36	TSS200	Island	9.3	2.89E−03	0.236
cg24041556	chr19:10736059	SLC44A2	TSS200	Island	9.1	2.15E−05	0.110
cg22257574	chr9:135754383	C9orf98	TSS200	Island	9.0	1.97E−05	0.110
cg23005885	chr15:90543450	ZNF710	TSS1500	Island	8.9	6.20E−04	0.169
cg05726756	chr17:46608288	HOXB1	TSS200	Island	8.6	1.67E−03	0.206
cg12782180	chr7:127880932	LEP	TSS1500	Island	8.5	9.78E−04	0.184
cg04675542	chr5:150284416	ZNF300	TSS200	Island	8.4	3.42E−03	0.244
cg10134527	chr6:33283015	TAPBP	TSS1500	Island	8.4	3.19E−03	0.241
cg23387569	chr12:58120011	LOC100130776	TSS200	Island	8.4	3.39E−04	0.155
cg17205324	chr14:23835595	EFS	TSS1500	Island	8.3	1.46E−04	0.133
cg24402300	chr19:55591437	EPS8L1	TSS1500	Island	8.2	2.02E−03	0.216
cg18081258	chr14:21494161	NDRG2	TSS1500	Island	8.2	1.05E−03	0.187
cg00730561	chr10:102279703	SEC31B	TSS200	Island	8.1	4.82E−04	0.162
cg17791651	chr1:38513489	POU3F1	TSS1500	Island	8.0	1.31E−04	0.133
Hypomethylated CpG sites							
cg03462171	chr16:1664488	CRAMP1L	TSS200	Island	− 8.2	2.30E−03	0.223
cg11648730	chr5:92907151	FLJ42709	TSS1500	Island	− 6.4	1.72E−03	0.207
cg04558166	chr1:210001279	C1orf107	TSS200	Island	− 4.0	1.75E−03	0.209
cg25472897	chr8:145560555	SCRT1	TSS1500	Island	− 3.1	1.53E−03	0.201
cg17612948	chr5:110427863	WDR36	TSS200	Island	− 3.0	1.70E−03	0.207
cg21665057	chr3:196295764	WDR53	TSS1500	Island	− 2.4	2.42E−03	0.225
cg12683173	chr7:69063404	AUTS2	TSS1500	Island	− 1.4	3.30E−03	0.242
cg04872557	chr1:76190008	ACADM	TSS200	Island	− 1.0	1.96E−03	0.215

### Chromosomal distribution of the DMCs

To further explore the methylation profile, we investigated the chromosome distribution of DMCs. Results showed that hypermethylated CpG sites were located at chromosomes 1, 6, 11, and 17 (proportion > 6%, Fig. 1d) and hypomethylated CpG sites were located at chromosomes 1, 2, 6, 7, and 11 (proportion > 6%, Fig. 1e).

**Fig. 2:**
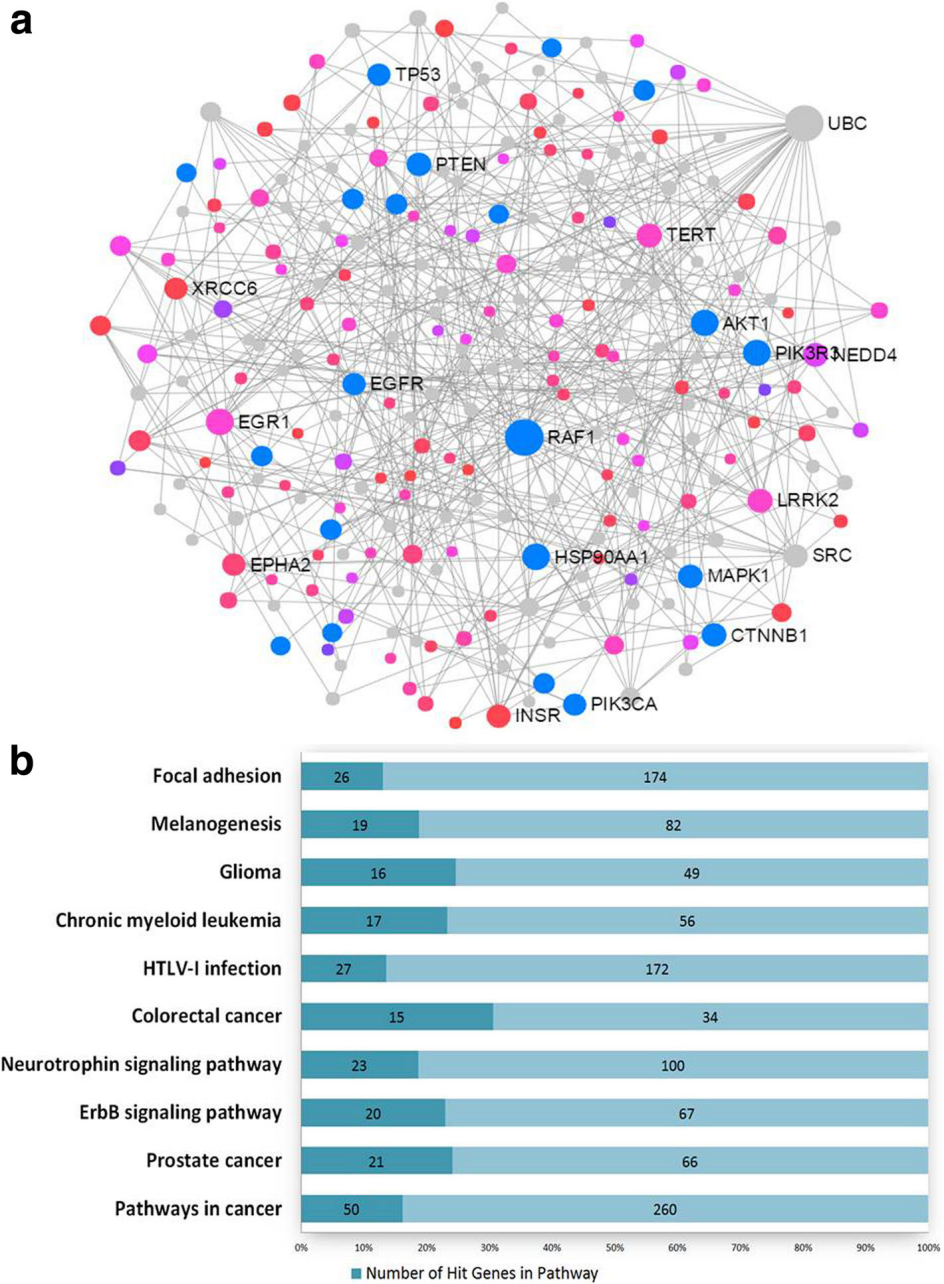
Protein-protein interaction analysis. **a** A subnetwork composing of 247 nodes and 403 edges was generated using methylated genes. Blue dots represent the genes involved in prostate cancer; red and pink dots represent the seeds (methylated genes) according to the different *P* values; the gray dots represent the proteins which were closely interacted with the seeds, and the circle size represents the node degree. **b** The pathway enrichment analysis shows the subnetwork is mainly enriched in cancer pathways (*P* < 0.0001)

Methylation variations of hypermethylated DMCs and hypomethylated DMCs were found mainly distributed on chromosomes 1, 6, and 11, suggesting that the DNA methylation alterations in these chromosomes were correlated with the body weight changes in prostate patients. Furthermore, we compared the distribution of the DMCs (hyper- and hypomethylated, separately) with the distribution of all evaluated CpG sites based on their relation to nearest gene regions (Fig. 2f, Additional file 4: Table S2) or their relation to CpG islands (Fig. 2g, Additional file 5: Table S3) using *χ*^2^ test. The results showed that hypermethylated CpGs are mainly located at TSS1500 (transcription start sites 1500), IGR (intergenic region), N-shore, and S-shore, and hypomethylated CpGs are mostly located at the gene body and open sea.

### Functional enrichment analysis of significantly obesity-associated DMCs

To investigate the potential biological relevance of the significant DMCs, we further filtered 483 DMCs (distributed within 413 genes) from a total of 5526 DMCs according to their locations at both the gene promoter and CpG island (Additional file 6: Table S4). Four hundred seventy-five of the 483 DMCs (representing 404 genes) were hypermethylated. Functional enrichment analysis of the hypermethylated genes showed that these genes were enriched for biological processes, such as pattern specification process, neuron differentiation, neuron fate specification, and negative regulation of phosphate metabolic process (adjusted *P* value < 0.05, Additional file 7: Table S5), as well as molecular functions, such as neuropeptide receptor activity and sequence-specific DNA-binding RNA polymerase II transcription factor activity (adjusted *P* value < 0.1, Additional file 8: Table S6). KEGG pathway enrichment analysis showed that hypermethylated genes were involved in signaling pathways regulating pluripotency of stem cells, fatty acid metabolism, basal cell carcinoma, non-alcoholic fatty liver disease (NAFLD), and AMPK signaling pathway (*P* value < 0.05, Additional file 9: Table S7).

We mapped the 404 hypermethylated genes to the STRING database and generated a protein-protein interaction (PPI) network by the NetworkAnalyst. The largest subnetwork was identified to include 247 nodes (genes) and 403 edges (Fig. 2a). In the network, the size of the nodes was based on their degree values and the color of nodes was based on their *P* values. This network contained 118 seed genes from the DMCs, and the enrichment pathway analysis showed that the genes of the subnetwork were mostly involved in the pathways of prostate cancer and other cancers (Fig. 2b, Additional file 10: Table S8, adjusted *P* value < 0.05). Particularly, the gene *UBC* (ubiquitin C) was found to be a hub connecting with many other nodes in the network, suggesting that the gene may play important biological roles in the PPAT of obese PCa patients.

### Selected genes with multiple methylated CpG sites

**Fig. 3:**
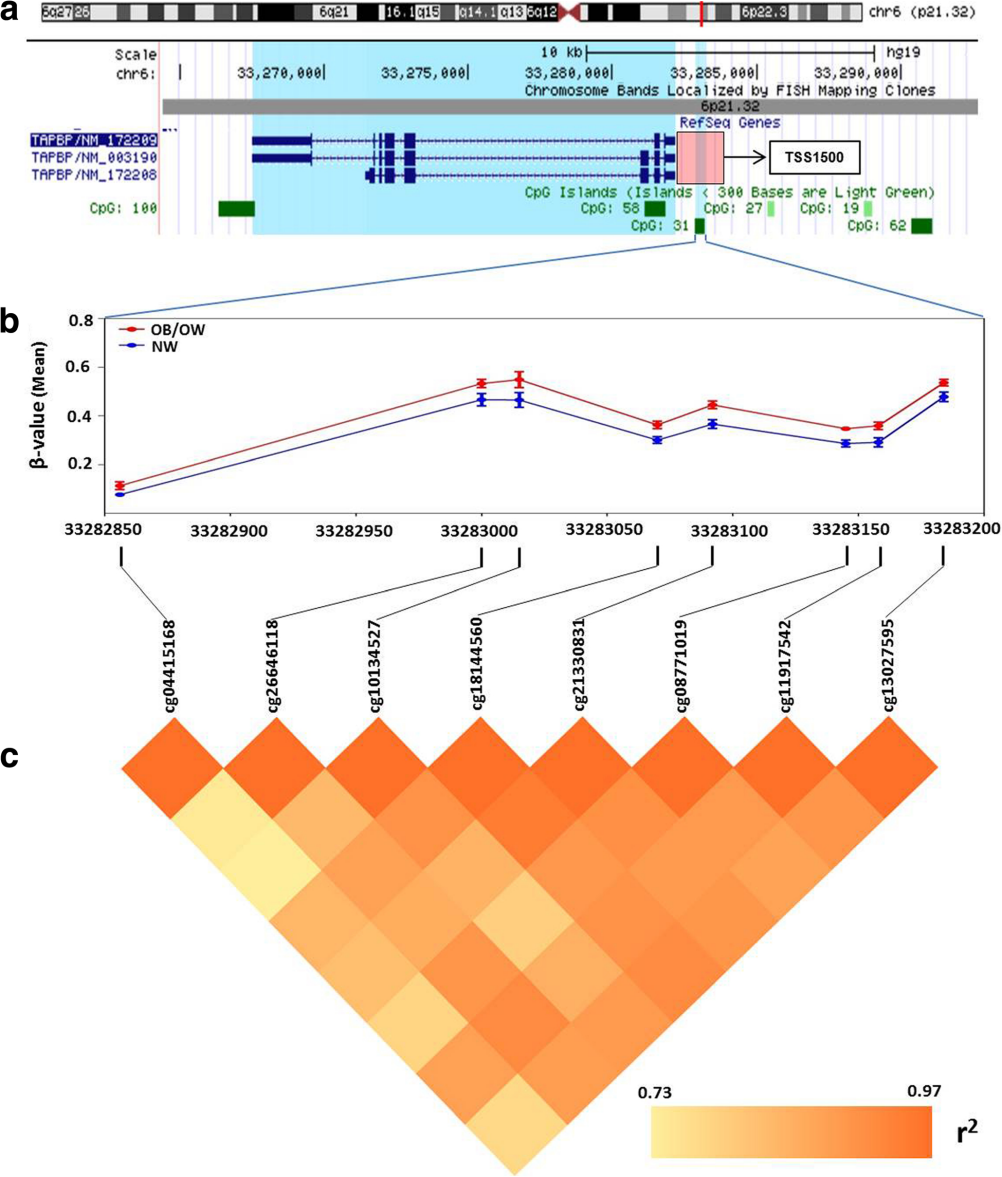
Visualization and analysis of hypermethylated CpG sites in TAPBP promoter. **a** The chromosome positions of hypermethylated CpG sites show that DMCs are located at chr6 (p21.32), which is in the region of TSS1500 (pink square) of TAPBP and at the location of CpG island 31. **b** Methylation levels of eight CpG sites in PPAT from OB/OW and NW PCa patients have shown a significant difference. **c** Correlation analysis shows strong correlation (Spearman correlation coefficient *r*^2^ 0.73~ 0.97) between the eight hypermethylated CpG sites based on the mean *β* value difference of individual probes

In order to explore repression of genes by DNA methylation modifications, we selected genes which had multiple hypermethylated CpG sites (the number of methylated CpG sites ≥ 2, in at least one of the sites with a mean difference of *β* > 3% and an adjusted *P* value < 0.25) (Additional file 1: Figure S1 and Additional file 11: Table S9). A total of 38 genes with 100 differentially methylated CpG sites were selected, which included *TAPBP*, *RUNX3*, *CPT1B*, *CPT1C*, *MOGAT3*, *WNT2*, and *AIRE* (Additional file 11: Table S9). Notably, the promoter region of *TAPBP* (TAP-binding protein) had eight hypermethylated CpG sites in the promoter (Fig. 3a), which were significantly more methylated in the OB/OW than those in the NW groups (Fig. 3b), with a mean difference of *β* value greater than 5% (Additional file 10: Table S8). Spearman’s rank correlation showed strong association (*r*^2^ = 0.73–0.97) of the eight hypermethylated CpGs in the *TAPBP* promoter with their methylation levels (Fig. 3c). Pathway analysis of these genes revealed enrichment for fatty acid metabolism, PPAR signaling pathway, glucagon signaling pathway, AMPK signaling pathway, glycerolipid metabolism, basal cell carcinoma, antigen processing and presentation, ECM receptor interaction, and insulin resistance (adjusted *P* value < 0.25) (Additional file 12: Table S10).

### Differential methylated regions analysis

Ten DMRs were identified (*P* < 0.01) in obesity PPAT samples compared to normal weight controls (Table 3). The size of the DMRs varied from 161 to 1287 bp. Noteworthy, four out of the ten DMRs were discovered on chromosome 6. Eight regions were located in genes, and two were in the intergenic region. Four regions were in the gene promoter of *FAM104A*, *C17orf80*, *HOXA4A*, and *TAPBP*.

**Table 3:** Differentially methylated regions (DMR) in PPAT of obese/overweight PCa patients compared to normal weight controls

DMR	Chr	Start–end (bp)	Size (bp)	*P* value	FDR	Located gene	DMCs*	Relation to CpG island
1	6	30,038,791–30,039,801	1010	5.11E−05	2.07E−02	RNF39	37(0)	Island
2	6	29,648,161–29,649,084	923	1.54E−03	2.08E−01	ZFP57^**#**^	22(0)	Open sea
3	17	71,228,123–71,228,832	709	3.10E−03	2.86E−01	FAM104A	4(3)	Island
4	17	71,228,123–71,228,832	709	3.10E−03	2.86E−01	C17orf80	10(8)	Island
5	12	42,720,006–42,720,167	161	5.56E−03	2.86E−01	PPHLN1	4(0)	Island
6	6	31,650,735–31,651,158	423	5.59E−03	2.86E−01	MIR4646^**#**^	16(0)	Island
7	7	27,169,674–27,170,961	1287	5.66E−03	2.86E−01	HOXA4	17(11)	Island
8	6	33,282,736–33,283,145	409	5.87E−03	2.86E−01	TAPBP	18(18)	Island
9	20	57,463,763–57,464,129	366	6.74E−03	2.86E−01	GNAS	15(0)	Island
10	16	86,546,938–86,547,322	384	7.03E−03	2.86E−01	FOXF1	4(0)	Shore

#The DMR is located at the intergenic region

*Chr* chromosome

*The number in the bracket is the quantities of DMCs located at the promoter (TSS200 and TSS1500) regions

### Association analysis between DNA methylation and mRNA expression

Increased DNA methylation of promoter in CpG islands was obviously linked to gene transcriptional silencing [26]. Therefore, we related hypermethylated CpG sites in PPAT with genes showed decreased gene expression level from our previously generated mRNA expression data [4]. DNA methylation of 16 CpG sites, corresponding to 14 genes, was associated with significantly decreased transcripts in OB/OW group (*P* value < 0.05) (Table 4). The Spearman’s rank correlation analysis showed that eight of the 14 genes have significantly negative association (*P* value < 0.05) between the methylation profiles and the gene expression profiles of these genes (Table 4). The repression genes were mainly involved in metabolic pathways (Additional file 13: Table S11, adjusted *P* value < 0.25), such as *MOGAT1* (glycerolipid metabolism), *FADS1* (fatty acid metabolism and biosynthesis of unsaturated fatty acids), and *PCYT2* (glycerophospholipid metabolism). The mRNA expression level of *FADS1* was significantly decreased in the PPAT of obese with prostate cancers in our previous study using qRT-PCR [4]. Besides these, GO enrichment analysis showed that these genes are functionally related to receptor binding (neuropeptide receptor binding, dopamine receptor binding, and insulin receptor binding) and enzyme activity (acid phosphatase activity, metallocarboxypeptidase activity, and acylglycerol *O*-acyltransferase activity) (Additional file 14: Table S12, adjusted *P* value < 0.25).

**Table 4:** Genes hypermethylated in promoters with significantly decreased gene expression

Gene symbol	DNA methylation			Gene expression			Correlation analysis	
	Probe ID	DNAm *β* diff. (%)	Adjusted *P* value (< 0.25)	Probe ID	FC	*P* value (< 0.05)	Spearman’s rank correlation coefficient	*P* value (< 0.05)
UCN	cg20442078	5.6	0.17	8051061	− 1.12	3.61E−02	− 8.42E−01	2.23E−03
CCHCR1	cg00160818	1.9	0.17	8124868	− 1.14	1.73E−02	− 7.45E−01	9.21E−03
CRB3	cg14782015	4.3	0.20	8025041	− 1.13	1.84E−02	− 7.21E−01	1.21E−02
AGBL4	cg21834207	3.2	0.13	7915971	− 1.17	1.29E−02	− 6.73E−01	1.97E−02
INSL3	cg10174482	4.2	0.13	8035345	− 1.13	4.94E−02	− 6.36E−01	2.72E−02
ANKRD30B	cg21293934	11.2	0.21	8069499	− 1.17	2.24E−02	− 6.08E−01	3.11E−02
FADS1	cg16213375	3.6	0.16	7948612	− 1.8	9.55E−04	− 5.88E−01	4.01E−02
PAPL	cg18481683	2.3	0.24	8028570	− 1.19	1.45E−02	− 5.52E−01	5.21E−02
MOGAT1	cg12678667	4	0.15	8048725	− 1.28	3.87E−02	− 4.67E−01	8.91E−02
PPP1R1B	cg09762778	5	0.12	8006865	− 1.27	4.74E−02	− 4.67E−01	8.91E−02
PRUNE2	cg00390775	4.4	0.15	8161884	− 1.31	1.91E−02	− 3.82E−01	1.39E−01
CIDEA	cg18309817	1.8	0.18	8020211	− 1.32	3.21E−02	− 2.97E−01	2.03E−01
PCYT2	cg19583655	6.2	0.21	8019280	− 1.26	6.38E−04	− 1.88E−01	3.04E−01
SCUBE1	cg07697597	1.7	0.23	8076586	− 1.23	9.03E−03	− 4.24E−02	4.59E−01

*FC* fold change, *DNAm β diff.* DNAm *β* difference

## Discussion

This pilot study revealed significant differences of DNA methylation profiles between the PPATs from OB/OW versus NW PCa patients. Variations in global DNA methylation demonstrated that excess adiposity played an important role in DNA methylation level of PPAT tissues in prostate cancer patients, which provide an opportunity to explore the effect of obesity on PPAT epigenetic modification and subsequently on prostate cancer. These findings reported for the first time in PPAT depot are in concordance with previous works reporting that excess adiposity and BMI activate DNA methylation in adipose tissue [27–29]. Thus, considering the present understanding of the potential causal relationship between excess adiposity and cancer [30], diabetes [11], and cardiovascular disease [31], our results provide methylated candidate genes, which might foster research on the potential biological mechanisms underlying epigenetic regulation of PPAT by excess adiposity and prostate cancer.

Given that DNA methylation of CpGs located at promoters and islands are associated with gene transcription silencing, we performed a strict filtering of DMCs and explored the biological functions of all promoter hypermethylated genes, aiming to find the critical methylated CpGs in the PPAT between the obese and normal weight PCa patients. Bioinformatic analysis showed that the enriched pathways were mostly involved in metabolic disorders, particularly fatty acid degradation and glycerolipid and choline metabolism. These pathways are known to mediate the pro-tumoral effect of white adipose tissue in tumors, thus contributing to tumorigenesis and metastasis [32, 33], particularly in prostate cancer [5]. Findings from other oncological models highlight excess adiposity-associated impact in methylation markers known to associate with potential effect in the cancer microenvironment (e.g., aromatase, prostaglandin E_2_ receptor in breast cancer) [34, 35]. Obesity has also been shown to associate with methylation of cancer-related genes (E-cadherin, p16, and RAR-β(2)) directly in malignant breast cells [36, 37].

**Fig. 4:**
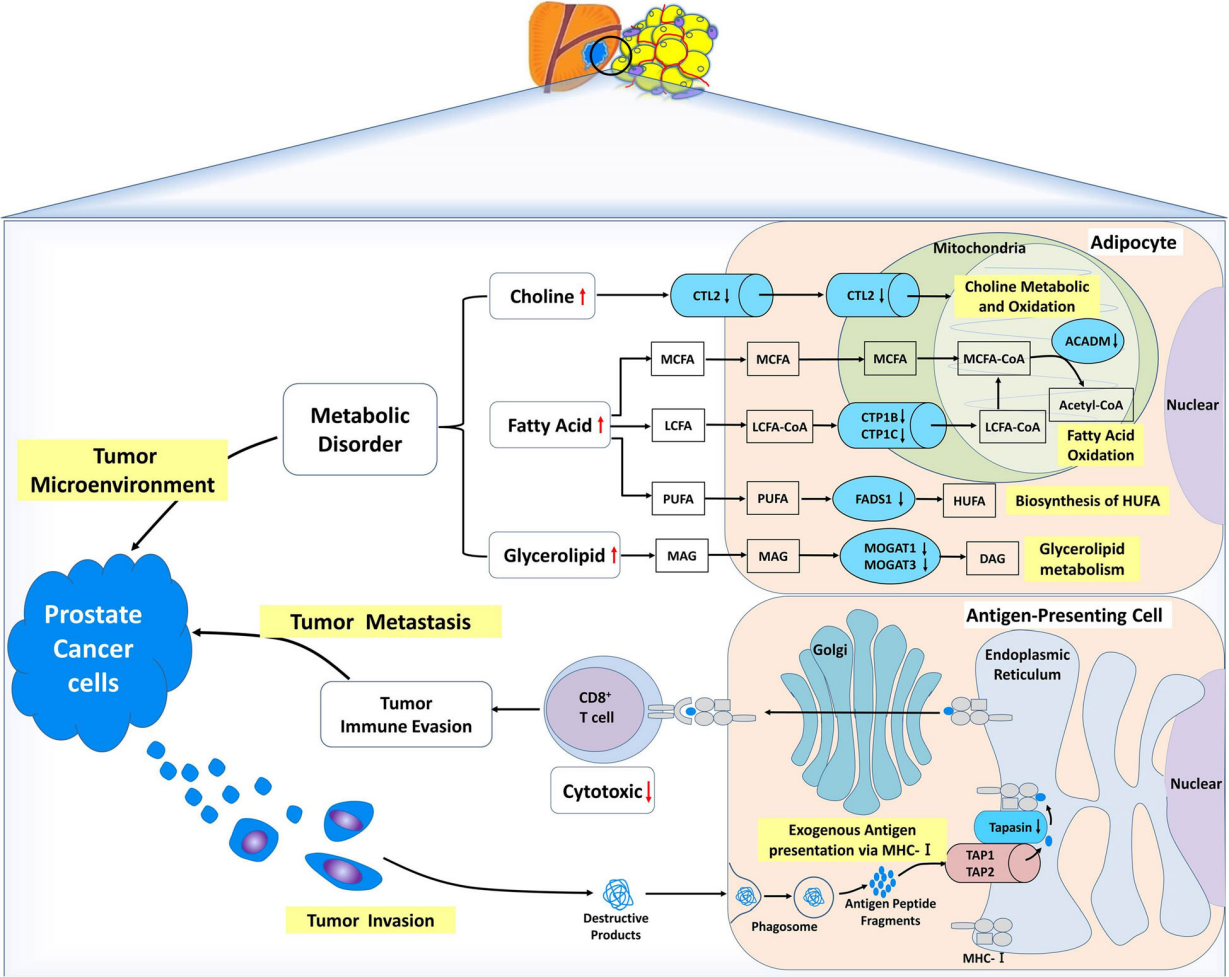
Proposed mechanisms with differentially methylated genes from PPAT of OB/OW prostate cancer patients. Hypermethylated genes in periprostatic adipose tissue of patients with increased adiposity might contribute towards the modulation of prostate tumor microenvironment. The genes that might be related to tumor microenvironment include choline transporter-like protein 2 (*CTL2*, which was a rate-limiting step of choline metabolism by transporting extracellular choline into cell and mitochondria), carnitine palmitoyltransferase 1B and 1C (*CPT1B* and *CPT1C*, which encode the rate-limiting enzymes of long-chain fatty acid β-oxidation by controlling transportation of long-chain fatty acyl-CoAs from cytoplasm across outer mitochondria membrane), medium-chain-specific acyl-CoA dehydrogenase (*ACADM*, which catalyzes the initial step of medium-chain fatty acid β-oxidation in mitochondria), fatty acid desaturase 1 (*FADS1*, which was correlated with fatty acid metabolism by catalyzing polyunsaturated fatty acid biosynthesis), monoacylglycerol *O*-acyltransferases 1 and 3 (*MOGAT1* and *MOGAT3*, which catalyze the formation of diacylglycerol by transferring fatty acyl-CoA to 2-monoacylglycerol), which contributes to metabolic disorder in adipose tissue by regulating the metabolism of lipid, choline, and glycerolipid. Another gene with hypermethylated promoter, *TAPBP* (transporter associated with antigen processing (TAP) transport protein), could influence tumor supervision of immune cells in PPAT by altering tumor antigen presentation process from TAP to MHC class I in endoplasmic reticulum and result in tumor metastasis and cancer progression. The black downward arrows represent the promoter hypermethylated genes (in blue containers), and the red arrows represent the possible consequence of these methylated genes. LCFA long-chain fatty acid, MCFA media-chain fatty acid, PUFA polyunsaturated fatty acid, HUFA high unsaturated fatty acid, MAG monoacylglycerol, DAG dionoacylglycerol

Pathway enrichment analysis showed a strong association between promoter hypermethylation of *CPT1B*, *CPT1C*, *ACADM*, and *FADS1*, with fatty acid metabolism. *CPT1B* (carnitine palmitoyltransferase 1B) and *CPT1C* (carnitine palmitoyltransferase 1C) genes encode rate-limiting enzymes in fatty acid degradation and play critical roles in long-chain fatty acid (LCFA) β-oxidation by controlling transportation of long-chain fatty acyl-CoAs from the cytoplasm across the outer mitochondria membrane [38]. Maple et al. reported that increased methylation of specific CpGs in the *CPT1B* promoter was correlated with decreased *CPT1B* transcripts in the skeletal muscle after lipid oversupply in severe obesity, which resulted in obese individual’s incapacity to increase fat oxidation, contributing to metabolic inflexibility [39]. Although the biochemical function of *CPT1C* has been verified to be necessary for the regulation of energy homeostasis in *CPT1C* knockout mouse brain [40], the study of *CPT1C* methylation was absent. *CPT1B* and *CPT1C* were previously reported to be highly expressed in the muscle, brain, and many other normal tissues including adipocytes [41]. Taken together, these findings suggest that methylation of specific CpG sites in the *CPT1B* and *CPT1C* promoters likely result in gene expression silencing, thus consequently contributing to fatty acid accumulation in adipocytes by decreasing long-chain fatty acid β-oxidation in the mitochondria (Fig. 4).

*LCFA* and *ACADM* genes (aliases MCAD, medium-chain acyl-CoA dehydrogenase) coding for metabolic enzymes presented increased methylation in the PPAT of the OB/OW group. *ACADM* is the critical enzyme of the initial step of β-oxidation and controls the medium-chain fatty acid (MCFA) metabolism by catalyzing the dehydrogenation of medium-chain Acyl-CoA, which is the common middle product of MCFA and LCFA, in the mitochondria. Mutations in *ACADM* cause MCAD deficiency, which resulted in fatty acid oxidation disorder leading to disease or infantile death [42–44]. Greco et al. [45] reported inverse association between *ACADM* transcript abundance with fat content in the human liver. Our findings suggest that the hypermethylated *ACADM* found in the PPAT of OB/OW PCa patients might fail to generate medium-chain acyl-CoA β-oxidation and result in MCFA and LCFA accumulation in adipose tissue, providing a favorable tumor microenvironment for PCa cell aggressiveness (Fig. 4). Additional functional studies are required to confirm this assumption.

The hypermethylation of the *FADS1* (fatty acid desaturase 1) promoter, whose transcriptional activity was significantly decreased in OB/OW PCa patients in agreement with our previous study [4], has been described as correlated with polyunsaturated fatty acid (PUFA) metabolism by catalyzing the biosynthesis of highly unsaturated fatty acids (HUFA) from the catalysis of dihomo-gamma-linoleic acid (DGLA, 20:3 n-6) and eicosatetraenoic acid (ETA, 20:4 n-3) desaturation, in order to generate arachidonic acid (AA, 20:4 n-6) and eicosapentaenoic acid (EPA, 20:5 n-3) [46]. Genetic variants in the *FADS1* and *FADS2* gene clusters have been associated with altered (n-6) and (n-3) PUFA metabolism [47, 48], whereas metabolic disorder in PUFA exerted effects on PCa by mediating the formation of eicosanoid inflammatory mediators (prostaglandins, leukotrienes, thromboxanes, and lipoxins), angiogenesis, immune cell regulation, and membrane structure and function [49, 50]. These results illustrated that the epigenetic modifications of *FADS1* may play important roles in the regulation of fatty acid metabolic genes on PPAT in response to excess adiposity (Fig. 4).

Besides abnormal fatty acid metabolism, DMC-related genes identified in our study were also correlated with glycerolipid metabolism. *MOGAT1* and *MOGAT3* encode the monoacylglycerol *O*-acyltransferase (MOGAT) and catalyze the formation of diacylglycerol (DAG) from monoacylglycerol (MAG), which is the precursor of phosphatidylcholine, phosphatidylethanolamine, and triacylglycerol (TAG), by transferring fatty acyl-CoA to 2-monoacylglycerol [51]. While human *MGAT1* (aliases for MOGAT1) is involved in intestinal dietary fat absorption and TAG synthesis in the liver, its function in adipose tissue has yet to be elucidated. The expression of *MGAT1* was increased in the liver of diet-induced obese mice with nonalcoholic fatty liver disease (NAFLD), but, interestingly, there was increased DAG accumulation and no inflammatory injury reduction in hepatocytes after *MGAT1* knockdown. Similarly, *MOGAT3* was mostly expressed in the human intestine and liver and maintained a significant DGAT (diacylglycerol *O*-acyltransferase) activity. Although results indicate that the metabolic mechanism of lipid regulation by *MGAT1* and *MOGAT3* was altered, evidence of association between lipid metabolic disorders caused by aberrant expression of MGAT1/MOGAT3 and PCa are lacking. Our data indicate the methylation of *MOGAT1* and *3* genes in PPAT may play important roles in response to excess adiposity by modulating glycerolipid metabolism (Fig. 4).

Choline metabolic disorder might be caused by epigenetic regulation of *SLC44A2* (solute carrier family 44 member 2), which encodes choline transporter-like protein 2 (CTL2) and is mainly expressed on blood plasma and mitochondrial membrane of different organisms and cell types. This transporter is a rate-limiting step in choline metabolism by transporting extracellular choline into cell and mitochondria. Choline is essential for synthesizing membrane phospholipid and neurotransmitter acetylcholine and used as a donor of methyl groups via choline oxidized in mitochondria [52]. The choline transporter has been associated with choline metabolic disorders, thus playing an important role in regulating immune response, inflammation, and oxidation [53, 54]. Concordantly, abnormal choline metabolism emerged as a metabolic hallmark, associated with oncogenesis and tumor progression in prostate cancer and other malignancies [55–57]. The increased uptake of choline by the cancer cell was important to meet the needs of phosphatidylcholine synthesis [58]. We hypothesize that hypermethylated *SLC44A2* in adipocytes might be associated with lower uptake and oxidation of extracellular choline, resulting in choline accumulation in PPAT extracellular media (Fig. 4) and increasing the availability of choline for PCa cell metabolism.

Besides metabolic modifications, altered immune regulation pathways were also enriched in DMC-related genes. *TAPBP* (alias tapasin) encodes a transmembrane glycoprotein, which mediates the interaction between MHC class I molecules and a transport protein TAP (transporter associated with antigen processing), being responsible for antigen processing and presentation. This mechanism occurs via mediating TAP to translocate endo/exogenous antigen peptides from the cytoplasm into the endoplasmic reticulum and deliver the antigen peptides to MHC class I molecules. The cancer cell’s survival depends on successful escape to immune surveillance. Loss of MHC class I has been described as a major immune evasion strategy for cancer cells. Downregulation of antigen-presenting MHC class I pathway in tumor cells was a common mechanism for tumor cells escaped from specific immune responses, which can be associated with coordinated silencing of antigen-presenting machinery genes, such as *TAPBP* [59]. Cross-presentation is the ability of certain antigen-presenting cells to take up, process, and present extracellular antigens with MHC class I molecules to CD8^+^ T cells. This process is necessary for immunity against most tumors. Recent studies revealed that *TAPBP* is a major target for cancer immune evasion mechanisms and decreased *TAPBP* expression in cancer was associated with reduced CD8^+^ T cell-mediated killing of the tumor cells, lowered immune responses, and enhanced tumor metastases via downregulation of antigen presentation the MHC class I pathway [60, 61]. Our results showed that *TAPBP* promoter hypermethylation in the PPAT of obese PCa subjects likely reduced the expression or activity of *TAPBP*, downregulating tumor cell’s antigen presentation of immune cells in PPAT, leading to impaired CD8^+^ T cell activation (Fig. 4). This indicates that methylation of *TAPBP* might be a mechanism by which prostate cancer cells escape the immune surveillance and provide an appropriate microenvironment for tumor aggressiveness, allowing prostatic cancer cells’ transfer, spread, and growth. The significant DMR identified with eight DMCs located in the *TAPBP* promoter further supported its role in prostate cancer.

From the PPI analysis, the network which was connected through ubiquitin C is characterized, suggesting *UBC* played a significant biological function with the methylated genes in PPAT between OB/OW and NW patients and somehow was correlated with the methylation. Ubiquitin is much known with the functions including roles in protein degradation, DNA repair, cell cycle regulation, kinase modification, and cell signaling pathways [62]. Recent reports expressed that the ubiquitin-proteasome system was associated with the progression and metastasis of prostate cancers [63, 64]. And long-term silencing of the *UBC* was found to be correlated with DNA methylation at the promoters [65]. Additional studies are needed to clarify whether the protein network for methylated genes impacts prostate cancer and if this difference is associated with ubiquitin C.

Although we present the first report on periprostatic adipose tissue methylation profile in association with excess adiposity measured by BMI, our results should be interpreted in the context of several potential limitations. This study is limited by small sample size, even though representative groups of OB/OW and NW are likely to be selected following the strict inclusion/exclusion criteria and between-group match by clinicopathological and demographic variables. Although we matched patients by clinicopathological characteristics between adiposity groups, tobacco smoking was more frequent among OB/OW compared with NW patients. Actually, albeit we cannot exclude an effect of smoking status on the presumably adiposity-associated findings presented herein, due to a known effect of tobacco on overall DNA methylation, data from previous reports indicate that methylation profiles are tissue-specific [66, 67] and that adiposity-associated DNA methylation occurs independently of tobacco smoking [68, 69]. Future studies will benefit from the confirmation of these results in larger sample sizes, determination of correspondence to matched prostate tumor methylation patterns, investigation of interactome at the interface between tumor and PPAT, and prospective investigations on the value of PPAT epigenetic modifications on cancer recurrence and survival. Future validation and replication are important to establish the accuracy and generalizability of the reported associations.

In summary, we observed differences in PPAT methylation between NW and OB individuals at several loci known to be involved in the metabolism of choline (*SLC44A2*), fatty acids (*CPT1B*, *CPT1C*, *ACADM*, *FADS1*), and glycerolipid (*MOGAT1*, *MOGAT3*) and in the regulation of exogenous tumor antigen presentation (*TAPBP*). These findings suggest a relationship of adiposity status with the methylation profile, which ultimately modulates tumor microenvironment and may influence PCa behavior.

## Conclusions

In this preliminary study, we report DNA methylation changes in PPAT underlying the association between excess adiposity and PCa. Whole epigenome methylation profiling of PPAT of PCa patients revealed significant differences in OB/OW versus normal weight subjects. Epigenetic imprinting in association with excess adiposity expressed the methylated modifications in genes functionally related with lipid metabolism and immune function, which could ultimately contribute to an unfavorable tumor microenvironment and decreased immune surveillance for prostate tumors. This association analyses provided us novel insights into how prostate cancer patients with excess adiposity differ from those of patients with normal weight in epigenome. Findings from this study warrant confirmation in PPAT samples from larger number of patients.

## Additional files

Additional file 1: **Figure S1.** Research flowchart. Whole research flowchart. NW normal weight, OB/OW obese/overweight, BMI body mass index, PPAT periprostatic adipose tissue, QC quality control, DMCs differentially methylated CpG sites, DMRs differentially methylated regions, Limma linear models for microarray and RNA-seq analysis data using *R*, GO gene ontology, KEGG Kyoto Encyclopedia of Genes and Genomes, PPI protein-protein interaction network. (JPEG 128 kb)

Additional file 2: **Table S1.** Differentially methylated CpG sites in PPAT between obese/overweight PCa patients and normal weight controls. The table shows 5526 DMCs in PPAT between obese/overweight PCa patients and normal weight patients, which were identified by using the “Limma” method. (XLSX 663 kb)

Additional file 3: **Figure S2.** Heatmap of differentially methylated CpG sites between the PPAT of OB/OW PCa and NW PCa patients. The graphical display of hierarchical clustering for DMCs. The selected CpGs are those with FDR < 0.25 and beta difference between obesity and normal weight group larger than 10%. (JPEG 1797 kb)

Additional file 4: **Table S2.** Distribution of differentially methylated CpG sites in relation to the nearest gene regions. The table shows the distribution of DMCs according to the relation to the nearest gene regions. (XLSX 11 kb)

Additional file 5: **Table S3.** Distribution of differentially methylated CpG sites in relation to CpG islands. The table shows the distribution of DMCs according to the relation to CpG islands. (XLSX 13 kb)

Additional file 6: **Table S4.** Differentially methylated CpG sites located at both gene promoters and CpG islands. This table shows the 483 DMCs which locate at both gene promoters and CpG islands. (XLSX 76 kb)

Additional file 7: **Table S5.** GO biological process analysis of promoter hypermethylated genes. GO biological process analysis for 404 promoter hypermethylated genes. (XLSX 18 kb)

Additional file 8: **Table S6.** GO molecular function analysis of promoter hypermethylated genes. GO molecular function analysis for 404 promoter hypermethylated genes. (XLSX 13 kb)

Additional file 9: **Table S7.** Pathway enrichment analysis of promoter hypermethylated genes. Pathway enrichment analysis for 404 promoter hypermethylated genes. (XLSX 11 kb)

Additional file 10: **Table S8.** Pathway enrichment analysis of the genes included in PPI networks. Pathway enrichment analysis for methylated genes and related genes included in PPI networks. (XLSX 13 kb)

Additional file 11: **Table S9.** Selected genes with multiple hypermethylated CpG sites in PPAT with obese/overweight. The table shows the 38 selected genes which have multiple hypermethylated CpG sites. (XLSX 24 kb)

Additional file 12: **Table S10.** Pathway enrichment analysis of the selected genes with multiple hypermethylated CpG sites. Pathway enrichment analysis for the 38 selected genes which have multiple hypermethylated CpG sites. (XLSX 13 kb)

Additional file 13: **Table S11.** Pathway enrichment analysis of the overlapping genes. Pathway enrichment analysis for the 14 overlapping genes. (XLSX 13 kb)

Additional file 14: **Table S12.** GO molecular function analysis of the overlapping genes. GO molecular function analyses for the 14 overlapping genes. (XLSX 13 kb)

## Abbreviations

AA: Arachidonic acid
ACADM: Aliases MCAD, medium-chain acyl-CoA dehydrogenase
BH: Benjamin and Hochberg
BMI: Body mass index
CPT1B: Carnitine palmitoyltransferase 1B
CPT1C: Carnitine palmitoyltransferase 1C
CTL2: Choline transporter-like protein 2
DAG: Diacylglycerol
DGAT: Diacylglycerol *O*-acyltransferase
DGAT2: Diacylglycerol *O*-acyltransferase 2
DGEs: Differential gene expressions
DGLA: Dihomo-gamma-linoleic acid
DMCs: Differentially methylated CpG sites
DMRs: Differentially methylated regions
EPA: Eicosapentaenoic acid
ER: Endoplasmic reticulum
EWAS: Epigenetic-wide Association Studies
FADS1: Fatty acid desaturase 1
FDR: False discovery rate
GO: Gene ontology
GWAS: Genome-wide Association Studies
HUFA: Highly unsaturated fatty acid
KEGG: Kyoto Encyclopedia of Genes and Genomes
LCFA: Long-chain fatty acids
limma: Linear models for microarray and RNA-seq data
MAG: Monoacylglycerol
MCFA: Medium-chain fatty acid
MGAT1: Aliases for MOGAT1
MHC: Major histocompatibility complex
MOGAT: Monoacylglycerol *O*-acyltransferase
MOGAT1: Monoacylglycerol *O*-acyltransferase 1
MOGAT3: Monoacylglycerol *O*-acyltransferase 3
NAFLD: Nonalcoholic fatty liver disease
PCa: Prostate cancer
PPAT: Periprostatic adipose tissue
PPI: Protein-protein interaction analysis
PSA: Prostate-specific antigen
PUFA: Polyunsaturated fatty acid
QC: Quality control
SLC44A2: Solute carrier family 44 member 2
TAG: Triacylglycerol
TAP: Transporter associated with antigen processing
TAPBP: TAP binding protein
TSS: Transcription start site
TSS1500: 1500 bp upstream of the transcription start site
TSS200: 200 bp upstream of the transcription start site
UBC: Ubiquitin C
