# Supplementary figures and images for: Epigenome-wide DNA methylation profiling of periprostatic adipose tissue in prostate cancer patients with excess adiposity—a pilot study
Source: Clin Epigenetics. 2018 Apr 17;10:54. doi: 10.1186/s13148-018-0490-3 (PMC5904983; doi:10.1186/s13148-018-0490-3)

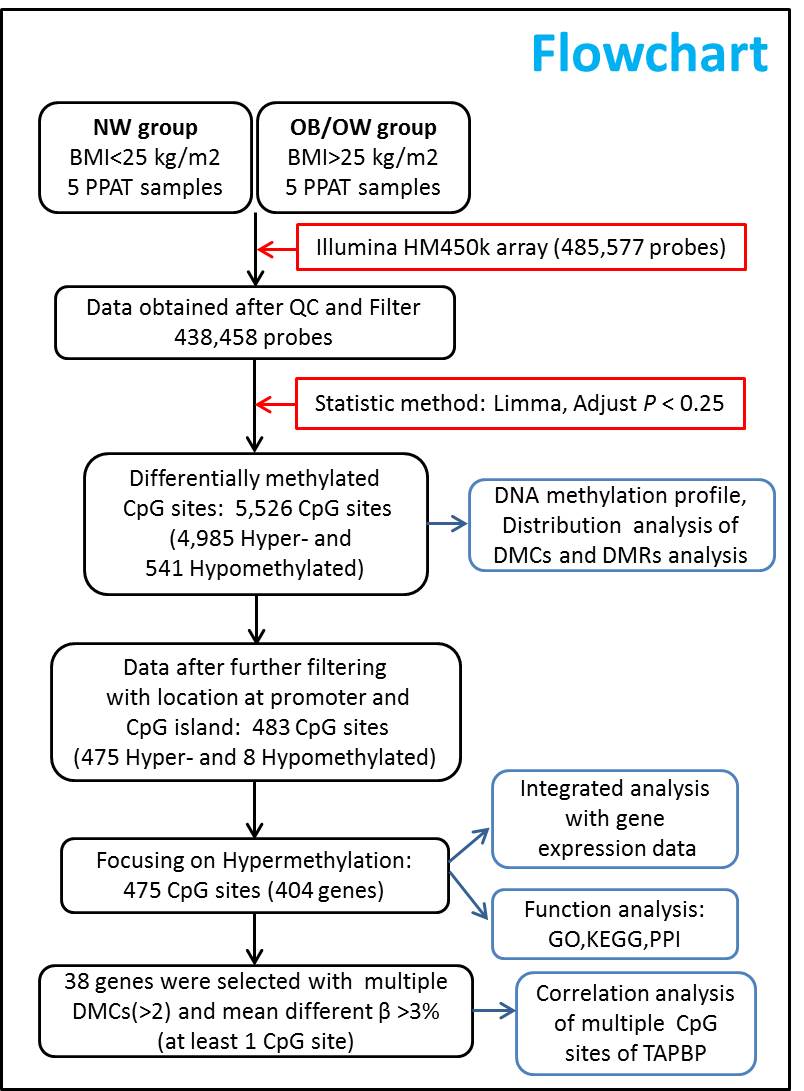

Supplement: Supplementary file 1 — Figure S1. Research flowchart. Whole research flowchart. NW normal weight, OB/OW obese/overweight, BMI body mass index, PPAT periprostatic adipose tissue, QC quality control, DMCs differentially methylated CpG sites, DMRs differentially methylated regions, Limma linear models for microarray and RNA-seq analysis data using R, GO gene ontology, KEGG Kyoto Encyclopedia of Genes and Genomes, PPI protein-protein interaction network. (JPEG 128 kb) [file 13148_2018_490_MOESM1_ESM.jpg]

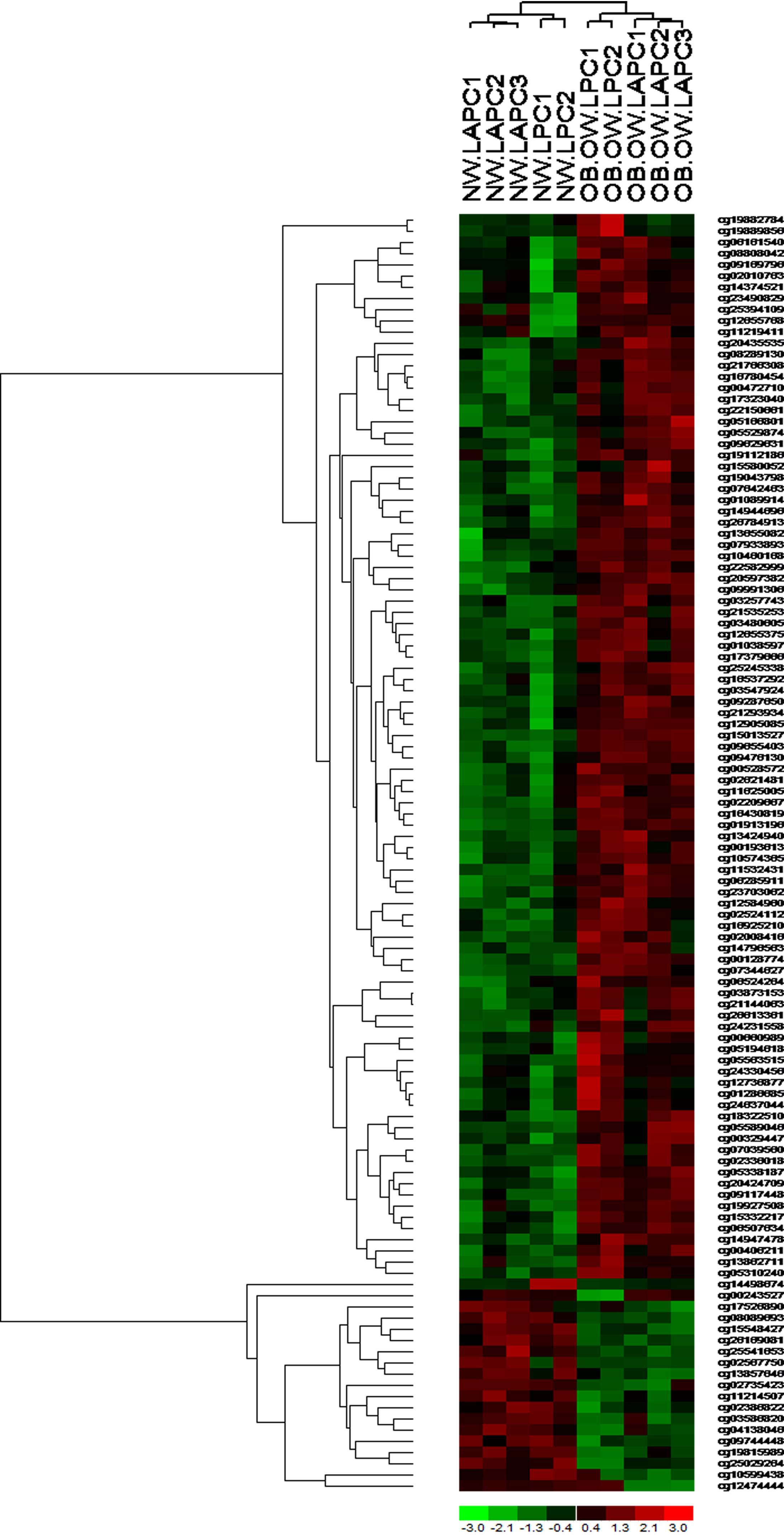

Supplement: Supplementary file 3 — Figure S2. Heatmap of differentially methylated CpG sites between the PPAT of OB/OW PCa and NW PCa patients. The graphical display of hierarchical clustering for DMCs. The selected CpGs are those with FDR < 0.25 and beta difference between obesity and normal weight group larger than 10%. (JPEG 1797 kb) [file 13148_2018_490_MOESM3_ESM.jpg]
